# Prefrontal Cortex and Somatosensory Cortex in Tactile Crossmodal Association: An Independent Component Analysis of ERP Recordings

**DOI:** 10.1371/journal.pone.0000771

**Published:** 2007-08-22

**Authors:** Yixuan Ku, Shinji Ohara, Liping Wang, Fred A. Lenz, Steven S. Hsiao, Mark Bodner, Bo Hong, Yong-Di Zhou

**Affiliations:** 1 Department of Neurosurgery, Johns Hopkins University, Baltimore, Maryland, United States of America; 2 Krieger Mind/Brain Institute, Johns Hopkins University, Baltimore, Maryland, United States of America; 3 Department of Neuroscience, Johns Hopkins University, Baltimore, Maryland, United States of America; 4 Department of Biomedical Engineering, Johns Hopkins University, Baltimore, Maryland, United States of America; 5 The Institute of Cognitive Neuroscience, East China Normal University, Shanghai, People's Republic of China; 6 The Music Intelligence Neural Development Institute, Costa Mesa, California, United States of America; 7 Tsinghua University, Beijing, People's Republic of China; University of Birmingham, United Kingdom

## Abstract

Our previous studies on scalp-recorded event-related potentials (ERPs) showed that somatosensory N140 evoked by a tactile vibration in working memory tasks was enhanced when human subjects expected a coming visual stimulus that had been paired with the tactile stimulus. The results suggested that such enhancement represented the cortical activities involved in tactile-visual crossmodal association. In the present study, we further hypothesized that the enhancement represented the neural activities in somatosensory and frontal cortices in the crossmodal association. By applying independent component analysis (ICA) to the ERP data, we found independent components (ICs) located in the medial prefrontal cortex (around the anterior cingulate cortex, ACC) and the primary somatosensory cortex (SI). The activity represented by the IC in SI cortex showed enhancement in expectation of the visual stimulus. Such differential activity thus suggested the participation of SI cortex in the task-related crossmodal association. Further, the coherence analysis and the Granger causality spectral analysis of the ICs showed that SI cortex appeared to cooperate with ACC in attention and perception of the tactile stimulus in crossmodal association. The results of our study support with new evidence an important idea in cortical neurophysiology: higher cognitive operations develop from the modality-specific sensory cortices (in the present study, SI cortex) that are involved in sensation and perception of various stimuli.

## Introduction

Recent monkey studies have shown evidence that cells in primary somatosensory cortex (SI) and secondary somatosensory cortex (SII) change their firing correlated with tactile unimodal working memory [1]–[4]. In a recent human study [5], it was shown that SI cortex retained a memory trace of the tactile stimulus for a short period. Further, cells in the somatosensory cortex of monkeys were shown to respond to task-related stimuli of more than one sensory modality in working memory tasks [6]–[8]. Crossmodal effects have also been observed in studies on neural mechanisms of attention in monkeys, in which firing changes in cells of somatosensory cortex were found in the crossmodal attention switch [9], [10], and in attention studies of humans, in which changes in early modality-specific sensory (visual, auditory, and somatosensory) ERP (event-related potential) components were detected [11], [12]. The above observations suggest that crossmodal links affect sensory-perceptual processes within modality-specific cortical regions [11].

In behavioral studies, it has been shown that viewing the stimulated body part can improve tactile discrimination at the stimulated site [13]–[15]. The visual–tactile improvement may be linked to modulations of neural activities in SI [15], [16] through the higher-level multimodal associative cortex [16]–[19], suggesting the involvement of both sensory and associative cortical areas in visual-tactile crossmodal associations.

In our previous study [20], we found that the amplitude of the ERP component N140 evoked by the tactile stimulus was increased when the subject expected a coming visual stimulus that had been paired with the tactile stimulus in comparison to this component evoked by the same tactile stimulus without crossmodal expectation. It has been suggested that the somatosensory N140 is generated by sources in multiple cortical areas, including frontal cortex and SII cortex [21]–[23]. By applying independent component analysis (ICA) in the present study to the EEG (electroencephalogram) data recorded in the unimodal and crossmodal tasks, we explored independent components (ICs) that represented neural activities in cortical areas. We found that the crossmodal modulation of the N140 represented the neural activities in somatosensory (SI, and possibly SII as well) and frontal cortical areas that cooperated with each other in crossmodal association in the tasks.

## Results

### Event-related potentials (ERPs)

**Figure 1:**
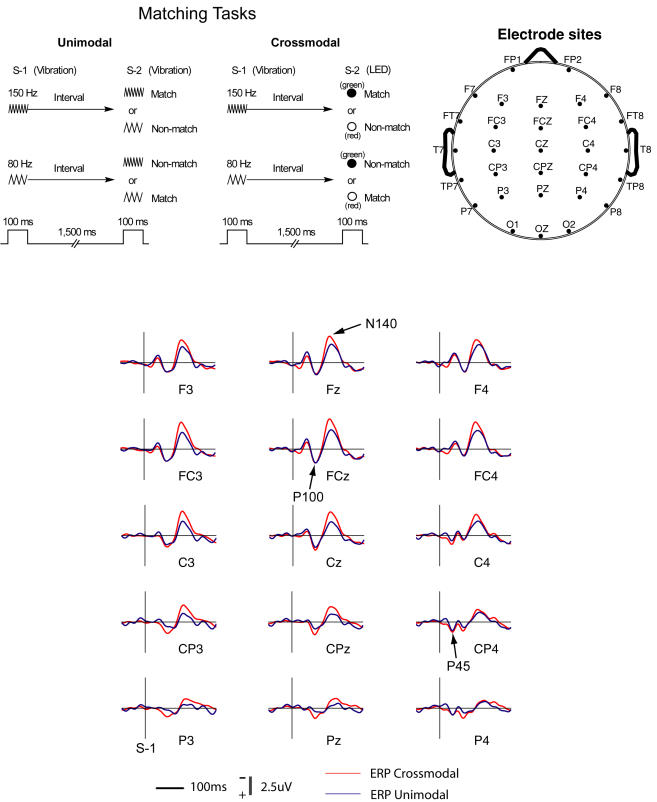
Tasks and scalp electrode distributions. *Upper-left:* Schematic description of delayed matching-to-sample tasks. In the unimodal matching task, stimulus-1 (S-1) is a tactile vibration (150 Hz or 80 Hz) delivered on the subject's left index fingertip. Stimulus-2 (S-2) is also a tactile vibration. In the crossmodal matching task, S-2 is a light (red or green) from a light-emitting diode (LED) presented in front of the subject at eye level. The green light matches high frequency and the red light, low frequency. *Upper-right:* a top view of scalp electrode distributions. Nose and ears are shown in the diagram. Ag-AgCl electrodes are in a standard arrangement for locations. *Lower:* Grand average ERPs recorded in performance of the matching tasks. ERP components P45, P100, and N140 are indicated by arrows. The ERPs are time-locked to the onset of stimulus-1 (S-1).

Results of the ERP analysis in this study were basically the same as the results from our previous study [20] since in the present study, the ERP data of 8 out of 10 participants were from that study. Figure 1 (*lower*) shows ERP components, P45, P100, and N140 at 15 electrodes. A three-way repeated measures multivariate analysis of variance (MANOVA) was performed for comparisons of amplitude and latency of the components. The within-subject factors were LR (left-right electrode locations), AP (anterior-posterior electrode locations), and Modality (crossmodal and unimodal). Amplitude of N140 recorded at those 15 electrodes was significantly affected by Modality (F = 12.8, df (Effect) = 1, df (Error) = 9, p<0.01) and AP (F = 16.9, df (Effect) = 4, df (Error) = 6, p<0.01), but not by LR. Latency of N140 was significantly affected by AP (F = 5.3, df (Effect) = 4, df (Error) = 6, p<0.001), but not by Modality or LR. Amplitude of P100 was significantly modulated by LR (F = 10.7, df (Effect) = 2, df (Error) = 8, p<0.01) but not by Modality or AP. Latency of P100 was significantly affected by LR (F = 8.3, df (Effect) = 2, df (Error) = 8, p<0.05), but not by Modality or AP.

### Independent components (ICs)

Thirty different independent components were found through ICA from each task of each subject. In comparison among them, 2 independent components across 2 tasks and 10 subjects showed consistent temporal activities and topographies of their coefficients of spatial projection to scalp electrodes. We defined those 2 ICs as IC-F (F: frontal) and IC-RS (RS: right somatosensory).

#### Topographies

**Figure 2:**
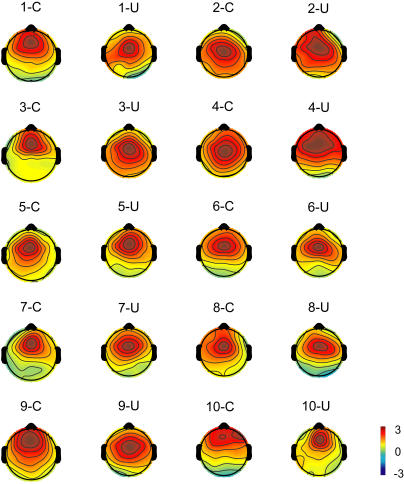
Topographic maps of an independent component (IC-F) located in frontal areas. Color-scale shows the value of the projection coefficient of the component. The topography of the IC-F is consistent across subjects (n = 10, indicated by numbers) and between tasks, unimodal (U) and crossmodal (C).

**Figure 3:**
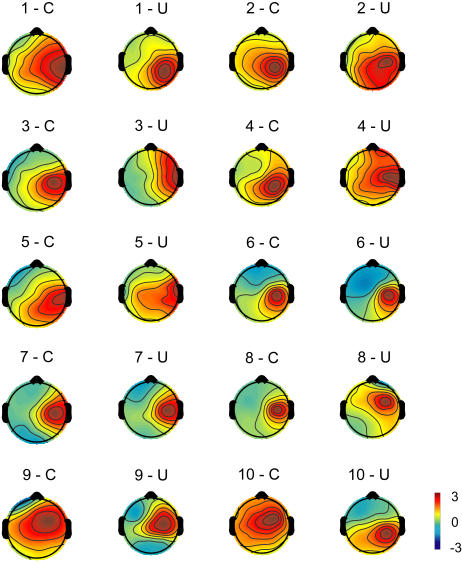
Topographic maps of an independent component (IC-RS) located in right somatosensory areas.

**Figure 4:**
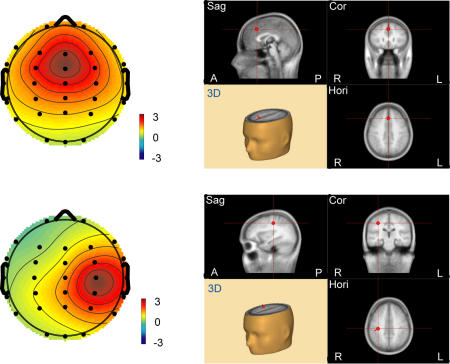
The average topography of IC-F (upper-left) and IC-RS (lower-left), and the corresponding BESA fitting dipole positions. The grand mean of the topographic maps is from 10 subjects across the tasks. Dipoles indicating the source of the components are located in medial prefrontal areas (IC-F, upper-right) and somatosensory areas (IC-RS, lower-right) respectively. Image views of the brain for each component are (clockwise from the top-left): sagittal (Sag), coronal (Cor), horizontal (Hori), and three-dimensional (3D). A: anterior; P: posterior; L: left; R: right.

The IC-F appeared to be active in prefrontal areas, and the IC-RS appeared to be active in right somatosensory areas. The topography of the IC-F is shown in Figure 2, and of the IC-RS is shown in Figure 3. Individual topographic maps were normalized by root mean square, and made the same polarities [24]. Topographies of both IC-F and IC-RS were apparently consistent across 10 subjects and the two tasks. Topographies of IC-F and IC-RS were averaged respectively across subjects and tasks (Figure 4), and the grand mean of the topographies of each IC was then submitted to BESA2000 to obtain the location of the IC-related dipole in the brain (Figure 4). The IC-F-dipole location was found to be around the medial prefrontal areas, anterior part of the midline of the brain (Talairach coordinates [25]: 0.5, 19.5, 43.4). This location was estimated to be in anterior cingulate cortex (ACC, area 32). IC-RS-dipole was estimated around the right primary somatosensory area (Talairach coordinates: 33.0,−22.5, 41.6, area 3).

#### Temporal activities

**Figure 5:**
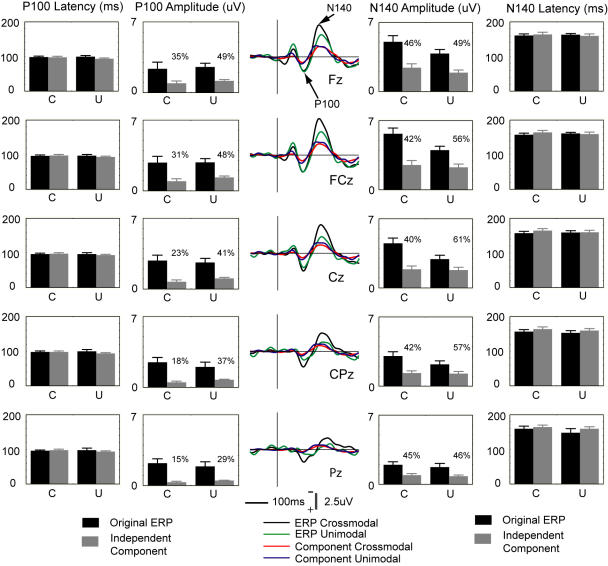
Comparisons of ERP components and IC projections. *Left:* Comparisons of latency and amplitude of ERP component P100 and IC-F projections. *Middle:* Grand averages of the original ERPs recorded from those midline electrodes, and grand average back-projections of the IC-F component to those electrodes. *Right:* Comparisons of latency and amplitude of ERP component N140 and IC-F projections. The percentage number indicates the proportion of potential that the IC-F contributes to the original ERP N140. C: crossmodal. U: unimodal. In bar graphs, the range of the ordinate for latency of P100 and N140 is 0–200 ms; the range of the ordinate for absolute values of amplitude of P100 and N140 is 0–7 uV. Error bars represent SEMs in this figure and other figures.

Temporal activities of two independent components, IC-F and IC-RS were analyzed. Back-projections of the IC-F showed waveforms with peaks similar to the original ERP components, P100 and N140 (Figure 5). A four-way repeated measures MANOVA (see Materials and Methods) showed no significant difference in latency of those two components between the IC-F back-projections and the original ERPs (P100: F = 3.3, df (Effect) = 1, df (Error) = 9, p = 0.103; N140: F = 0.2, df (Effect) = 1, df (Error) = 9, p = 0.691). Results of the amplitude analysis showed that substantial proportions of the original P100 and N140 were contributed by the IC-F. No significant difference was observed between modalities in IC-F contributions to the N140, although the significant difference between modalities in this component was shown in the original ERPs.

**Figure 6:**
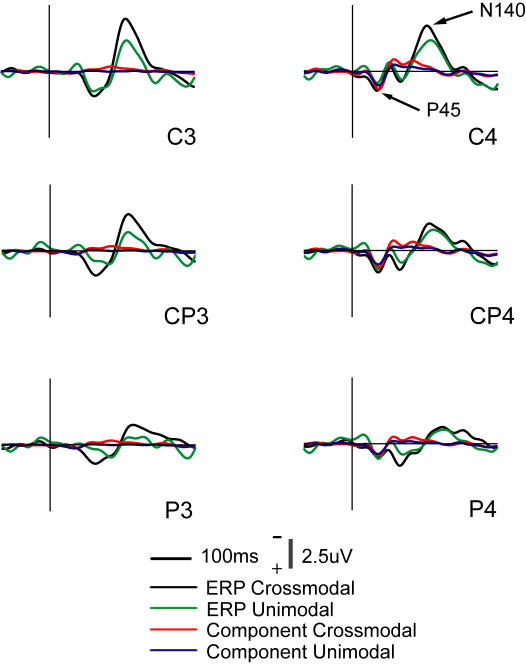
Grand averages of the original ERPs recorded at the electrodes (C4, CP4, and P4) contralateral to the tactile stimulus, and also at those (C3, CP3, and P3) ipsilateral to it. ERP component P45 is shown at those contralateral electrodes. Grand average back-projections of the IC-RS component to those electrodes are also shown, where the projections have the largest peaks. Note those ERP P45 peaks and IC-RS back-projections are similar in both latency and amplitude.

**Figure 7:**
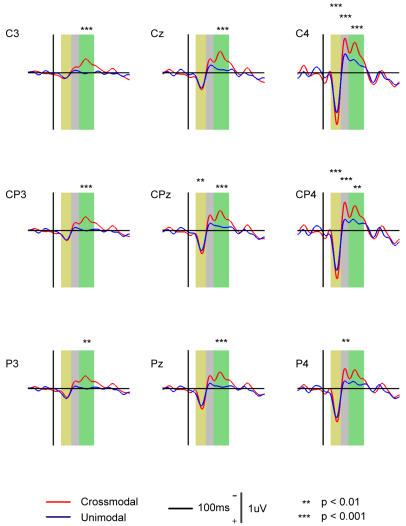
Grand average back-projections of the IC-RS. The significant difference in the projections at the electrodes between unimodal and crossmodal tasks are labeled with asterisks in three durations: 30∼70 ms (yellow), 70∼100 ms (gray), 100∼160 ms (green).

Back-projections of the IC-RS showed a component similar to the original ERP component P45 observed at three electrodes on the right side, contralateral to the tactile stimulus (Figure 6). No significant difference was observed between the IC-RS component and the ERP P45 in latency among the 9 electrodes. This IC-RS component was significantly affected among these 9 electrodes by AP (F = 12.1, df (Effect) = 2, df (Error) = 8, p<0.005) and LR (F = 33.6, df (Effect) = 2, df (Error) = 8, p<0.0005), but not by Modality (F = 4.3, df (Effect) = 1, df (Error) = 9, p = 0.07) although Post hoc test (Tukey HSD) showed that this component was significantly higher in the crossmodal task at several electrodes (figure 7).

Back-projections of the IC-RS in the ranges of 70∼100 ms, and 100∼160 ms were analyzed respectively. The peak amplitude in the duration of 70∼100 ms was significantly affected by AP (F = 17.0, df (Effect) = 2, df (Error) = 8, p<0.005) and LR (F = 19.1, df (Effect) = 2, df (Error) = 8, p<0.001), but not by Modality, and in the duration of 100∼160 ms the peak amplitude was also significantly affected by AP (F = 9.2, df (Effect) = 2, df (Error) = 8, p<0.01) and LR (F = 16.0, df (Effect) = 2, df (Error) = 8, p<0.005), but not by Modality. For both durations, interactions between AP and LR were significant (F = 6.9, df (Effect) = 4, df (Error) = 6, p<0.02). The results of Post hoc test (Tukey HSD) for differences in the amplitude between the two tasks are shown in Figure 7.

### Time-frequency representation (TFR), coherence, and Granger causality spectra

**Figure 8:**
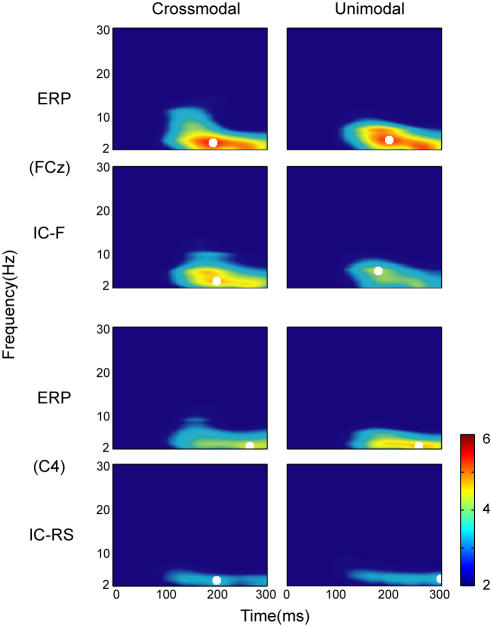
Time-frequency representation (TFR) for IC-F and the original ERPs at FCz (upper), and for IC-RS and the original ERPs at C4 (lower). Results are the average of all trials over 10 subjects and displayed in units of standard deviation of the baseline. Time zero is the onset of stimulus-1 in the tasks, crossmodal and unimodal. The peak frequency is indicated by a white square in each corresponding representation.

Power spectra for IC-F and the original ERPs at FCz, and for IC-RS and the original ERPs at C4 were analyzed across all subjects (Figure 8). At these two electrode sites, back-projections from IC-F and IC-RS showed highest amplitude respectively. Results indicated that in the time range of 100∼300 ms, independent components and ERPs showed activities mainly with frequency in the theta band (3–7 Hz).

**Figure 9:**
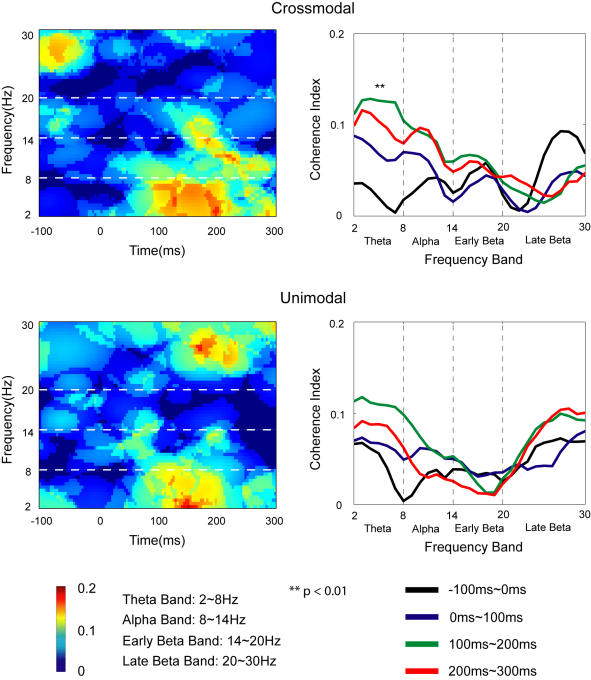
Average coherence between IC-F and IC-RS across subjects. *Upper*: Results in the crossmodal task; *Lower*: Results in the unimodal task. Time-frequency representation of coherence index is shown on the left side for both tasks. The coherence index across different frequency bands during different time durations is shown on the right side for the tasks. Post hoc (Tukey HSD) test shows that the theta-band coherence during 100∼200 ms is significantly different from the baseline (−100∼0 ms) in the crossmodal task.

Coherence between IC-F and IC-RS is indicated in Figure 9 (left side). A three-way repeated measures MANOVA (see Materials and Methods) showed no significant effects of any main factor (Modality, Duration, Frequency-Band). Post hoc test (Tukey HSD), however, showed in the crossmodal task the significantly stronger coherence in the theta band during 100∼200 ms after the onset of S-1 compared with the baseline (−100∼0 ms).

**Figure 10:**
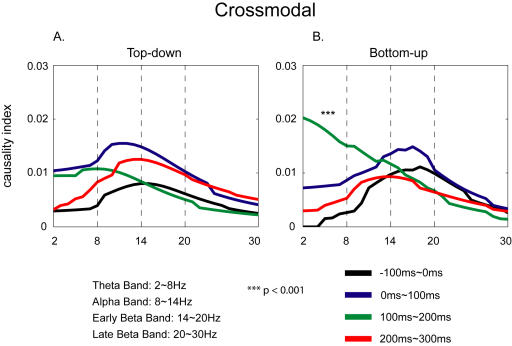
Granger causality analysis between IC-F and IC-RS for the crossmodal task. Post hoc (Tukey HSD) test shows that in the theta band the bottom-up connectivity in the period of 100∼200 ms is significantly stronger than that in the baseline (−100∼0 ms). *Top-down*: Granger causality from IC-F to IC-RS; *Bottom-up*: Granger causality from IC-RS to IC-F.

Granger causality spectra were obtained (see Materials and Methods) to test the direction of the connectivity between IC-F and IC-RS in the crossmodal task since significant coherence was observed between these ICs in the task. Results showed trends that the connectivity after the onset of the stimulus (0∼300 ms) was stronger than before the stimulus (−100∼0 ms) in the task (Fig. 10). The causality index (CI) was significantly affected by Frequency-Band (F = 10.5, df (Effect) = 3, df (Error) = 7, p<0.01), but not by other two factors (Modulation, Duration). The interaction between Modulation and Frequency-Band was marginal (F = 4.2, df (Effect) = 3, df (Error) = 7, p = 0.05). Post hoc analysis showed that, in general, the pre-stimulus CI was the smallest. For crossmodal bottom-up modulation (Fig. 10), CI in the theta band in the duration from 100 ms to 200 ms after the onset of S-1 was significantly (p<0.001) larger than that before the S-1 (−100∼0 ms).

## Discussion

ICA is a technique that has been successfully applied to human EEG studies in the last decade [24], [26]–[31]. ICA completely decomposes single-trial (or continuous) EEG data, separating the data into distinct information sources. By using this technique in data analysis, the multi-channel EEG can be decomposed into spatially fixed, temporally maximally independent components, and the scalp maps associated with some of these ICs resembled the scalp projections of synchronous activity inside the brain in a cognitive task [27]. Thus, when subjects perform behavioral tasks the ICs likely represent neural activities in those brain areas where they are located [24], [26], [27], [31], [32]. In the present study, the use of the ICA technique enabled us to find from the original EEG data two ICs (IC-F and IC-RS) that represented neural activities correlated with tactile working memory tasks, unimodal or crossmodal. This finding strongly suggests that cortical locations of those two ICs, medial prefrontal cortex and SI are involved in perception of the tactile stimulus and crossmodal associations in the task, and it may therefore provide us with a better understanding of the neural mechanism underlying the crossmodal working memory. The results of our study showed the beneficial application of the ICA technique that leads us to valuable findings that would not have been possible with the traditional ERP analysis.

Studies have shown that P100 and N140 are enhanced when attention is directed to the somatosensory stimuli, and are modulated by endogenous spatial attention as well [22], [33]–[40]. In the present study, the subject's attention was directed to the tactile stimulus (S-1) to detect the frequency of vibration. The level of attention was essentially the same in both tasks. Back-projections of IC-F showed its substantial non-differential (similar in both tasks) contributions to P100 and N140, indicating that medial prefrontal cortex, most likely ACC, was one of the major sources of these two ERP components, which represented neural activities of ACC in attention to the same tactile stimulus (S-1) in the tasks. This finding showed that the ACC was involved in attention on the tactile stimulus as early as 100 ms after the onset of the stimulus. The present finding is consistent with the findings of other studies showing that the ACC plays an important role in attention of various stimuli [23], [41]–[48].

The back-projection analysis showed that another independent component found in this study, IC-RS, was the main generator for the ERP component P45 that typically represents the neural activity in SI cortex evoked by the contralateral somatosensory stimulus [21], [35], [36], [49]. This suggested that changes in back-projection from the IC-RS represented neural activities of SI cortex in the task. The location of the IC-RS also supported the notion that the dynamic changes in IC-RS activity represented the changes in SI activity. Significant differences in IC-RS back-projection between the unimodal task and the crossmodal task were observed after the onset of the tactile stimulus, apparently because of the enhancement of IC-RS activity in the crossmodal task. The enhancement and the location of IC-RS strongly suggested that the crossmodal association between tactile and visual stimuli involved activities in the SI cortex as early as 100 ms after the onset of the tactile stimulus, or even earlier since P45 of the back-projection from IC-RS also showed trends toward differential reaction between the tasks. This new finding in the present study agrees with the findings in other studies that show participation of SI cortex in crossmodal association in monkeys [6]–[8], and in humans [15], [16].

In our previous study [20], we argued that the enhancement of N140 in the crossmodal task was unlikely to be due to attention, movement, or load of the task, but rather was related to crossmodal transfer of information between the tactile and the visual modalities in the task. The IC-F found in our present study had a sizeable contribution to the N140, but its contribution did not show any significant difference between the tasks. Although we were not able to locate an IC that was consistent across subjects and tasks in SII area because of the limitations of the ICA technique, it is a reasonable assumption that the significant difference in N140 between the tasks likely resulted from the activity in SII since SII has been shown to be another major source, in addition to the prefrontal source, to generate somatosensory N140 [22], [50]–[53]. Nonetheless, the possibility that other prefrontal areas contributed to the difference in N140 cannot be completely eliminated. It has also been suggested that P100-generators are located in the SII cortex [51], [54]–[57]. Therefore, the crossmodal modulation of both P100 and N140 generated by the subject's expectation of visual stimuli in the task may involve the change in neural activities in the SII cortex. The results of our study suggest that the crossmodal association may not only occur in association cortical regions, such as frontal cortex and posterior parietal cortex, but also occur in tactile modality-specific cortical regions, such as SI and SII cortex.

Studies have shown that the ACC is involved in attentional modulation of sensory processing in primary visual and primary auditory cortices (e.g., [47] ). The theta oscillation in ACC may play an important role in the attentional modulation [58]–[60]. Our coherence analysis indicated that in the crossmodal task, compared with the baseline period the coherence in theta range during the period of 100∼200 ms after the onset of the tactile stimulus was significantly increased between IC-F and IC-RS, showing that the activity in SI cortex may be synchronized with the activity in ACC in crossmodal association. This coherence between two areas suggested that ACC cooperated with SI cortex in attention and perception of the tactile stimulus under the influence of the crossmodal association. The Granger causality analysis of the coherence indicates that activities of ACC may be affected by SI cortex (bottom-up) as early as 100∼200 ms after the onset of the tactile stimulus.

In conclusion, modulation was observed in the present study on activities of somatosensory cortex in the crossmodal task. Although how tactile crossmodal association is processed in the somatosensory system is still not well understood, our study clearly shows that SI cortex (presumably SII cortex as well) participates in the task-related crossmodal association that has been suggested by previous monkey and human studies (e.g., [7], [16] ). In the process of crossmodal association, somatosensory cortex appeared also to cooperate with the higher level association cortex, the medial prefrontal cortex, in attention and perception of the tactile stimulus. Taken together, the results of our study support the idea with new evidence that higher cognitive operations develop from the modality-specific sensory cortices that are involved in sensation and perception of various stimuli [1], [61]–[68], and fit the concept of the perception-action cycle [69], [70] that describes the cortical neural dynamics of sensory-motor behaviors.

## Materials and Methods

Details of experimental procedures for behavioral tasks and EEG recording have been described previously [71].

### Participants

Twelve paid normal adult volunteers were recruited for the present study (10 men, 2 women, aged 19–47 years). Two participants were excluded because of excessive blinking or excessive muscle artifacts. Thus data from 10 subjects were collected and analyzed in the study. The data of 8 out of those 10 subjects were also used previously [20]. All participants signed informed consent. The protocols of the experiments were approved by the IRB of the Johns Hopkins School of Medicine.

### Stimuli and EEG recording

Experiments were carried out in a quiet, dimly lit room. Participants sat in a comfortable chair, facing a light-emitting diode (LED) about 1.5 m away in the center of the visual field at eye level. Visual stimuli (LED) comprised green or red light 100 ms in duration. Tactile stimuli were generated by a mechanical vibrator with frequency 80 Hz and 150 Hz, and delivered on the subject's left index finger-tip. During performance of tasks, participants placed their left hand on a supporter in their usual position to receive the tactile stimulus, and their right hand on another supporter to press two buttons as the response with their fingers, the index finger for left button; the middle finger for right button.

Electroencephalograms (EEG) were recorded by an EEG recording system (SynAmp, Neuroscan, Ltd Corp). Thirty-two Ag-AgCl scalp electrodes (Quick-Caps, Neuroscan) were arranged in standard locations (Guideline thirteen, American Clinical Neurophysiology Society, 2003). EEG signals from all of these electrodes were referenced to linked earlobes. The impedance of each electrode was kept below 5 kΩ. The electro-oculogram (EOG) was recorded for horizontal eye movements and for vertical eye movements. Signals of EEG (30 electrodes) and EOG (2 electrodes) were filtered (0.1–100 Hz band-pass), amplified, digitized (500 Hz sampling rate), and saved for off-line analysis.

### Behavioral tasks

The scalp-ERPs were recorded when participants performed a tactile-tactile delayed matching-to-sample task (unimodal task) or a tactile-visual delayed matching-to-sample task (crossmodal task). The subjects were instructed to focus on the LED throughout a recording session to avoid eye movement and eye blinking within any trial of the task. Trials that showed eye-blinks, excessive eye movements, or muscle artifacts were excluded from data analysis.

In the unimodal task, a complete trial contained a sequence of events (Figure 1 *upper*). A trial started with stimulus-1 (S-1), a 100-ms tactile vibration of either high (150 Hz) or low (80 Hz) frequency. After a delay of 1,500 ms, stimulus-2 (S-2), a 100-ms tactile vibration again (150 Hz or 80 Hz) was presented. During the delay, the subject was instructed to memorize the vibration frequency of S-1, and to expect S-2 that would match the frequency of S-1. The subject indicated at the end of the trial whether S-2 matched S-1 by pressing one of two buttons (e.g., left for match, right for nonmatch). The frequency of S-1 or S-2 was presented randomly from trial to trial to prevent the subject from getting any clue for performance. The intertrial interval between trials was chosen randomly in a range of 4–5 seconds. The subject's response time to S-2 was recorded.

In the crossmodal task (Figure 1 *upper*), the task sequence was identical to the unimodal task except that in this task S-2 was a visual cue (100 ms), a green or red LED associated with the tactile vibration. Associations between the tactile stimuli (S-1) and the visual stimuli (S-2) were assigned before the subject started performing the task (e.g., green associated with high frequency; red with low frequency) and counterbalanced across subjects. At the end of each trial, the subject indicated by pressing a button whether S-2 (LED) was associated with S-1.

### EEG data analysis

Original EEG data from which trials with eye-blinks, excessive eye movements, or muscle artifacts had been excluded were filtered with a digital zero-phase filter (Finite Impulse Response filter, pass band 2 to 30 Hz). Amplitude of an ERP component was calculated as the difference between its peak and the baseline (200 ms preceding the onset of S-1) mean value. Its latency was measured from S-1 onset to the peak. A three-way repeated measures MANOVA was performed for comparisons of amplitude and latency of the original ERP components. The within-subject factors of the analysis were left-right electrode locations (LR) (left, center, right–corresponding to electrode locations of 3, z, and 4), anterior-posterior electrode locations (AP) (frontal, frontocentral, central, centroparietal, parietal levels–electrode locations of F, FC, C, CP, and P), and Modality (crossmodal and unimodal).

### Independent component analysis

Analysis of EEG data recorded from 30 electrodes was performed by using Matlab 7.0 (Math Works, Natick, MA) and EEGLAB4.51(Swartz Center for Computational Neurosciences, La Jolla, CA; http://www.sccn.ucsd.edu/eeglab), a freely available open source software toolbox. BESA2000 (MEGIS, Graefeling, Germany) was also used to localize dipoles of independent components (ICs).

The filtered-EEG data (2∼30 Hz) that preserved theta, alpha, and beta band information [31] were used for the ICA study. The onset of S-1 in each trial was used as the task-event marker to separate a trial into a period before the onset and a period after the onset. In each trial, filtered-EEG of 1,500 ms (500 ms before the onset of S-1 and 1,000 ms after it) were extracted from the continuous EEG to form a data epoch. The mean value of EEG amplitude in the first 100 ms of the epoch was calculated from all trials of each task in each individual subject. To obtain the EEG data epoch for further processing, this mean value was then subtracted from each corresponding data epoch to reduce the influence of EEG variance across trials.

All data epochs were put together and submitted to infomax ICA [72], [73] that comes from the ICA families performing blind source separation. A 30×30 unmixing square matrix was found by using Infomax ICA. When this matrix was multiplied by the EEG data epochs, maximally, temporally independent activities were obtained. In this calculation of the independent activities, a weight change of 10e-6 together with iterations <800 were set as the stop criterion [24].

Let X denote the EEG data and M denote the unmixing square matrix. Then independent activities (S) are: S = MX. We can change the formula into X = M^−1^S. In this formula, one row of the matrix S represents the temporal activity of one IC, and the corresponding column of the matrix M^−1^ represents this IC's spatial pattern at the scalp electrodes. The back-projection of an IC at one electrode is obtained by multiplying the temporal activity of this IC with its coefficient of the corresponding spatial pattern at this electrode. EEG at one electrode can be considered as the sum of back-projections of all ICs at this electrode. The temporal independent activity and its corresponding spatial pattern together characterize an IC that may correlate with the activity of a neuronal clique. In the present study, we screened activities of ICs to determine potentially common temporal patterns of those ICs across all trials of each task and subjects, and we also visually screened topographies of ICs to assess their potentially common spatial patterns across tasks and subjects. ICs showing event (onset of S-1)-related activities consistently across trials of each task and subjects, and spatial topographies consistently across tasks and subjects, were selected in the screening. The spatial topographies may reflect the dipole activity, presumably caused by partially synchronous activities within certain cortical source patches that produce far-field potentials through volume conduction. The above process of selection resulted in identification of ICs for each subject and each task, which presumably reflected activities of neuronal cliques in certain cortical areas.

Grand average back-projections of the selected ICs to the scalp electrode sites were compared with the original scalp ERPs. The contribution of each IC to an original ERP component was assessed by calculating the proportion of the back-projection of the IC to that ERP component. Latency and amplitude of the components of both the original ERPs and the back-projections of ICs were statistically analyzed by performing a four-way repeated measures MANOVA with Modality, Type (ERPs, and IC's back-projected activity), AP, and LR as within-subject factors. The amplitude of the components of IC projections and ERPs was normalized by subtracting its corresponding baseline (200 ms preceding the onset of S-1) mean values before the statistical analysis.

The grand average topography across subjects and tasks of a selected IC was submitted to BESA2000 that uses a standard four-shell spherical head model (i.e., brain, cerebrospinal fluid, bone, and scalp) to find the location of the IC-related dipole (source model) in the brain. The dipole was derived in BESA2000 by fitting it iteratively to the averaged IC topography parameters until minimal residual variance was reached. In the present study, the values of residual variance lower than 10% were used as the threshold [74].

### Time-frequency representations (TFRs) and coherence

TFRs of ICs and ERPs from the electrodes that showed the largest IC back-projections were computed on single trials in the frequency range of 2∼30 Hz by using Hanning windowed short time Fourier transformation. The window had a fixed length of 250 ms, moving across every time stamp. The mean value of the windowed-period was taken away to avoid the variation of direct current. Zeros were then added after each windowed-period to make TFRs smoother across the Frequency axis. The ratio of the zero-pad to the windowed-period was 32. The TFRs were then normalized for each frequency by subtracting the baseline (200 ms before the onset of S-1) mean value, and dividing by the baseline standard deviation [75].

The coherence spectrum of the two independent components (ICs) was calculated by using:
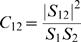
S_1_, S_2_ are the power spectra of the two ICs respectively. S_12 _is the cross spectrum between the two ICs. The value of coherence (C_12_) at frequency f ranges from 1, indicating maximum interdependence between the two ICs, down to 0, indicating no interdependence. Trials were shuffled 200 times to examine the significance of the coherence values (p<0.05).

The window length used in the coherence calculation was the same as those in calculation of the power spectrum. The ratio of the zero-pad to the windowed-period was 8. A three-way repeated-measures MANOVA was applied to compare mean coherence values among tasks, time durations, and frequency bands with Modality (crossmodal or unimodal), Duration (−100∼0 ms, 0∼100 ms, 100∼200 ms, 200∼300 ms, with the onset of S-1 as time 0) and Frequency-Band (Theta Band: 2∼8 Hz; Alpha Band: 8∼14 Hz; Early Beta Band: 14∼20 Hz; Late Beta Band: 20∼30 Hz) as the within-subject factors.

TFRs of ICs and ERPs, and coherence between ICs were also calculated with the window length of 500 ms. Results were similar to those obtained from the above analysis with the window length of 250 ms (see supplementary material, Figure S1 and Figure S2).

### Granger Causality Spectral Analysis

In order to examine the directional relationship between the two ICs, Granger causality spectral analysis [76], [77] was applied to evaluate the relative strength of influence. For each subject, the mean value of EEG of each trial was calculated and subtracted from the trial to get zero-mean stochastic process that is required for application of the autoregressive modeling. The multivariate autoregressive (MVAR) model was estimated with the 100-ms window for all trials in the time range from 100 ms before, to 300 ms after, the onset of S-1. The MVAR model of order m describes the data as:
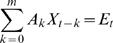
Where *E_t_* is a temporally uncorrelated residual error with covariance matrix *D*, and *A_k_* are 2×2 (2 ICs) coefficient matrices. Once the model coefficients *A_k_* and *D* are estimated, the spectral matrix can be written as:
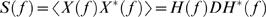
Where the asterisk denotes matrix transposition and complex conjugation, and 
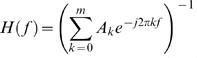
 is the transfer function of the system. In the present study, the optimal order for the MVAR model was determined by the Akaike Information Criterion (AIC) [78]. The order of 5 was selected because the AIC dropped monotonically with increasing model order up to 5. The Granger causality spectra were then calculated. The power at a specific frequency could be decomposed into an intrinsic part and a predicted part by other signals. The Granger causality at each frequency was thus defined by the ratio of predicted power to total power [77]. Causality Index was calculated by using:
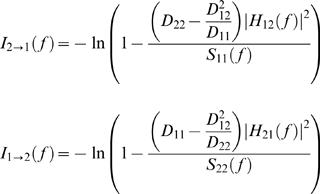
Where *D*_11_, *D*_22_, and *D*_12_ are elements of *D*, *S*_11_( f ) and *S*_22 _( f ) are the power spectra of channel 1 and channel 2 at frequency f respectively [77].

Trials were also shuffled 200 times to examine the significance. Granger causality spectra that were significant (p<0.05) were averaged across subjects. A three-way repeated-measure MANOVA was performed to compare causality values among time durations, and frequency bands with factors: Modulation (Top-down: direction of causality from IC-F to IC-RS; Bottom-up: IC-RS to IC-F), Duration, and Frequency Band.

## Supporting Information

Figure S1 Time-frequency representation (TFR) for IC-F and the original ERPs at FCz (upper), and for IC-RS and the original ERPs at C4 (lower). Results are the average of all trials over 10 subjects and displayed in units of standard deviation of the baseline. Time zero is the onset of stimulus-1 in the tasks, crossmodal and unimodal. The peak frequency is indicated by a white square in each corresponding representation. The window length in the analysis is 500 ms.
(1.84 MB EPS)

Figure S2 Average coherence results of IC-F and IC-RS across subjects. Upper: Results in the crossmodal task; Lower: Results in the unimodal task. Time-frequency representation of coherence index is shown on the left side for both tasks. The coherence index across different frequency bands during different time durations is shown on the right side for the tasks. Post hoc (Tukey HSD) test shows that the theta-band oscillation during 100 ms–200 ms is significantly different from the baseline (−100 ms–0 ms) coherence. The window length in the analysis is 500 ms.
(1.31 MB EPS)
